# The role of AXL and the *in vitro* activity of the receptor tyrosine kinase inhibitor BGB324 in Ewing sarcoma

**DOI:** 10.18632/oncotarget.2648

**Published:** 2014-11-15

**Authors:** Emmy D.G. Fleuren, Melissa H.S. Hillebrandt-Roeffen, Uta E. Flucke, D. Maroeska W.M. te Loo, Otto C. Boerman, Winette T.A. van der Graaf, Yvonne M.H. Versleijen-Jonkers

**Affiliations:** ^1^ Department of Medical Oncology, Radboud University Medical Centre, Nijmegen, the Netherlands; ^2^ Department of Pathology, Radboud University Medical Centre, Nijmegen, the Netherlands; ^3^ Department of Pediatric Hematology and Oncology, Radboud University Medical Centre, Nijmegen, the Netherlands; ^4^ Department of Radiology and Nuclear Medicine, Radboud University Medical Centre, Nijmegen, the Netherlands

**Keywords:** AXL, Gas6, Ewing sarcoma, BGB324, R428

## Abstract

New targets for Ewing sarcoma (ES) patients are urgently needed. Therefore, we investigated the expression and genetic aberrations of the oncogenic receptor tyrosine kinase (RTK) AXL in ES and determined the efficacy of AXL targeting on cell viability and migration. First, AXL and Gas6 (ligand) mRNA expression was determined by RT-PCR on 29 ES samples. Low, medium and high AXL mRNA expression was observed in 31% (*n = 9*), 48% (*n = 14*) and 21% (*n = 6*) of samples. Gas6 was abundantly present in all specimens. We next tested AXL protein expression immunohistochemically in 36 tumors (primary, post-chemotherapy, metastasized and relapsed samples) from 25 ES patients. Low, medium and high AXL protein expression was observed in 17% (*n = 6*), 19% (*n = 7*) and 36% (*n = 13*) of samples. In primary tumors (*n = 15*), high AXL expression correlated significantly with a worse overall survival compared to patients with lower expression (61 vs. 194 months, *p* = 0.026). No genetic aberrations were detected in the AXL RTK domain (*n = 29*). The AXL-inhibitor BGB324 affected viability (IC_50_ 0.79–2.13 μmol/L) and migratory potential of all tested ES cell lines *in vitro* (*n = 5–6*). BGB324 chemosensitized chemotherapy-resistant ES-4 cells to vincristine and doxorubicin. These data suggest that AXL is a potential novel, druggable therapeutic target in ES.

## INTRODUCTION

Ewing sarcoma (ES) is a highly aggressive type of primary bone cancer, predominantly affecting children and adolescents. Despite intensive surgery, radiotherapy and polychemotherapy, the final outcome in patients with metastatic disease has not improved impressively during the last decades and side-effects of treatment could be severe [1, 2]. This urges the need for novel, more specific treatment strategies [3]. One group of promising candidates of therapeutic targets are the receptor tyrosine kinases (RTKs). RTKs are membrane-bound proteins and are important regulators of cellular processes, such as cell growth, proliferation and survival. In addition to their pivotal role in normal physiology, several RTKs have been implicated in many human cancers [4]. More importantly, the targeting of RTKs proved to be a valid approach to inhibit growth of multiple tumor types. Although some RTKs have been implicated in ES, predominantly the Insulin-like Growth Factor-1 Receptor (IGF-1R), and more recently also MET and Anaplastic Lymphoma Kinase (ALK), the role of several other RTKs remains to be elucidated [5–8]. In this study, we investigated the role of AXL in ES.

AXL (or UFO) is a member of the TAM (Tyro3, AXL and MER) RTK family and plays an important role in various cellular processes, including survival, proliferation, migration, invasion and angiogenesis. Growth-arrest-specific protein 6 (Gas6) is the ligand with the highest affinity for AXL. Similar to other RTKs, AXL activation results in intracellular signal transduction involving phosphatidylinositol 3 (PI3)/Akt kinase and extracellular signal regulated kinase (Erk) pathways [9]. Moreover, various studies demonstrated the oncogenic potential of AXL, including a crucial role for AXL in the epithelial-mesenchymal transition (EMT), which is a key driver of metastasis, and mechanisms of drug resistance including both conventional chemotherapy as well as targeted therapies [10]. AXL overexpression and/or activation was demonstrated in a variety of human tumors, including several sarcomas [11–16]. AXL expression is also associated with metastasis in many cancer types, including osteosarcoma [9, 12, 17]. High AXL expression predicts a poor outcome in patients with myxoid liposarcoma and activated AXL was an independent predictor of a worse prognosis in osteosarcoma patients [12, 13]. Interestingly, AXL is also induced during acquired therapy resistance including against IGF-1R-targeted therapy in a rhabdomyosarcoma model [18]. There are some indications that AXL could be important in ES. For instance, gene expression profiling of 14 primary tumors from localized, nonmetastatic ES patients with clinical follow-up data revealed a poor prognosis signature group in which the *AXL* gene was one of many genes found to be significantly overexpressed [19]. In addition, a phosphoproteomics study identified tyrosine phosphorylated AXL in 1 out of 2 ES cell lines (RD-ES) [20].

Recently, the first-in class, highly selective AXL tyrosine kinase inhibitor BGB324 (R428) entered phase I clinical studies [21]. Encouraging first results of the phase Ia study were reported and phase Ib trials in solid and hematological malignancies are planned in the near future [22, 23].

The exact function of AXL in ES is, however, still unclear. We therefore examined AXL and Gas6 mRNA and AXL protein expression levels in a well-defined cohort of human ES specimens and correlated AXL protein expression levels to clinicopathological characteristics and patient outcome. Several tumor samples were examined for genetic AXL aberrations as well. The effect of the AXL inhibitor BGB324 was examined on ES cell viability, chemo-sensitivity and migration *in vitro* to explore the functional relevance of AXL-targeting in ES.

## RESULTS

### AXL and Gas6 expression in ES patients

**Figure 1:**
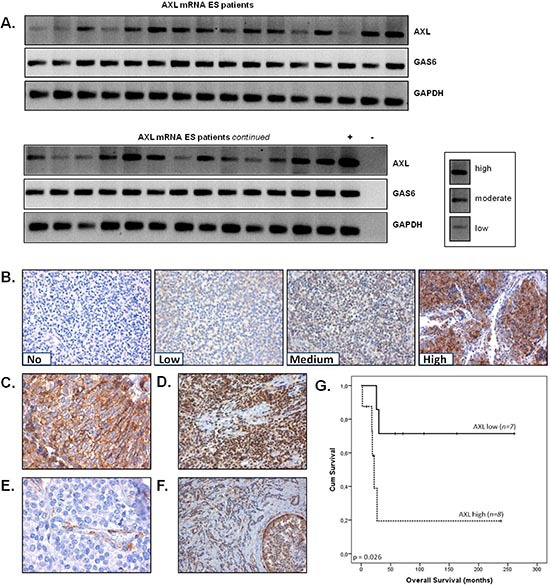
Expression and prognostic value of AXL in ES patients **(A)** AXL and Gas6 mRNA expression in ES specimens as determined by RT-PCR. mRNA expression frequency was evaluated semi-quantitatively (low, medium and high). GAPDH served as a loading control, the MDA-MB-231 breast cancer cell line as positive control (+) and MilliQ as negative control (−). **(B)** Representative images for AXL protein expression levels in ES specimens as determined by IHC. No (0), low (1), medium (2) and high (3) staining intensities are shown. All images are x200 magnification. **(C)** Example of apparent membranous AXL expression, x400 magnification. **(D)** Example of nuclear AXL expression, x400 magnification. **(E)** Example of AXL staining in the endothelial cells of a tumor blood vessel, x400 magnification. **(F)** AXL expression in AXL-positive BC tumor specimen, 200x magnification. All images (B-F) are haematoxylin counterstained. **(G)** Kaplan-Meier analysis: ES patients with the highest (score 3) level of AXL expression (AXL high; *n = 8*) in their primary tumor had a significantly worse OS compared to patients with lower (score 0, 1 and 2) AXL expression levels in the primary tumor (AXL low; *n = 7*) (*p* = 0.026).

AXL and Gas6 mRNA expression levels were analyzed in 29 fresh-frozen ES patient samples. Medium and high AXL mRNA expression was observed in 48% (*n = 14*) and 21% (*n = 6*) of ES samples, respectively. Low expression levels were detected in 31% (*n = 9*) of cases (Figure 1A). Gas6 was abundantly and identically present in all ES samples. These findings prompted us to further investigate AXL protein expression levels and target localization in a well-defined cohort of ES patients with clinical follow-up data.

AXL protein expression levels were immunohistochemically analyzed in 36 samples obtained from 25 ES patients, including 15 primary tumors (pre-treatment), 11 post-chemotherapy resections, 7 distant metastases and 3 local relapses (Table 1). Immunopositivity (defined as intensity score > 0) for AXL was detected in the majority of specimens (*n = 26*; 72%) and virtually all positive sections demonstrated protein expression in > 70% of tumor cells (Figure 1B). Low and medium AXL expression was observed in 17% (*n = 6*) and 19% (*n = 7*) of ES samples, respectively. However, the most outstanding samples were those with high AXL expression, which was observed in 36% (*n = 13*) of cases. AXL expression levels in these lesions were substantially higher than in samples with medium or lower AXL scores, and therefore seemed to be a distinct group. Also compared to a panel of 80 breast carcinoma (BC) samples stained in parallel, including triple-negative samples and selected positive BC tissue controls, considerably higher AXL expression was observed in ES samples (Figure 1F). AXL was predominantly localized in the cytoplasm, although some cases demonstrated apparent membrane-accentuated or nuclear AXL expression (Figure 1C and 1D). In some samples, vascular endothelial AXL expression was detected (Figure 1E).

**Table 1:** Patient characteristics

	Evaluable for AXLNo. (%)
**Total patients**	25 (36 samples)
**Sample origin**	
Primary tumor (pre-treatment)	15 (42%)
Post-chemotherapy resection	11 (31%)
Metastasis	7 (19%)
Relapse (local failure)	3 (8%)
**Gender**	
Male	9 (36%)
Female	16 (64%)
**Age at diagnosis** (years)	
Median (range)	14 (2 – 53)
≤ 14	14 (56%)
> 14	11 (44%)
**Tumor stage** (at diagnosis)	
Localized	22 (88%)
Metastatic	3 (12%)
**Tumor location**	
Extremity	10 (40%)
Pelvis	6 (24%)
Other	9 (36%)
**Translocation**	
EWS/FLI1	22 (88%)
EWS/ERG	2 (8%)
Other	1 (4%)
**Events**	
Metastasis	12 (48%)
Relapse	10 (40%)
Dead	14 (56%)
**Intervention**	
Chemotherapy	25 (100%)

In six ES specimens, both AXL mRNA and AXL protein expression levels were evaluated. There was a significant positive correlation between AXL mRNA and protein expression (Spearmans ρ, *p* = 0.013, *r* = 0.867, *n = 6*).

In the post-chemotherapy resection group, AXL expression was significantly lower compared to expression in the group of primary tumors (Table 2; *p* = 0.009). We could not confirm this finding in a paired analysis, because there were only three patients from which paired primary and post-chemotherapy resection specimens were scored.

**Table 2:** AXL expression levels in primary tumors and post-chemotherapy resections

Lesion origin	AXL IHC score (*n*, %)				
	0	1	2	3	*p*-value^1^
**Primary tumors** (*n = 15*)	1 (7%)	3 (20%)	3 (20%)	8 (53%)	0.009
**Post-chemotherapy resections** (*n = 11*)	7 (64%)	2 (18%)	0 (0%)	2 (18%)	

1 *p*-values are calculated using Fisher's exact test (contingency tables).

Kaplan-Meier (log-rank test) survival analysis was performed on the primary tumors (*n = 15*) to investigate a possible correlation between AXL expression and patient outcome. No, low, medium and high AXL expression was observed in 7% (*n = 1*), 20% (*n = 3*), 20% (*n = 3*) and 53% (*n = 8*) of primary tumors, respectively. Because samples with highest AXL expression (score 3) seemed to be a distinct group as described above and to increase statistical power for survival analysis, the primary tumor cohort was divided into two groups: low (scores 0–2) and high (score 3) AXL expression. Interestingly, high AXL expression correlated significantly with a worse OS compared to low expression (61 ± 37 vs. 194 ± 40 months, *p* = 0.026) (Figure 1G). There was no significant correlation between AXL expression and clinical characteristics including tumor stage, tumor location and age at diagnosis. Although some of these characteristics have been linked to a poor prognosis in ES in other studies, none of the characteristics correlated significantly with a poor OS in our primary tumor cohort (not shown). These findings suggest that AXL expression in the primary tumor is an independent prognostic marker of poor prognosis in ES, which was confirmed in a Cox multivariate regression analysis (*p* = 0.021)(Supplemental Table 1).

### Genetic AXL aberrations

Twenty-nine ES patients and six ES cell lines were examined for genetic alterations in mRNA encoding the AXL RTK domain. This domain was selected because it is the intracellular catalytic subunit of the receptor mediating signal transduction, and BGB324 specifically interferes with this domain [24]. However, no aberrations were observed.

### Targeting AXL in ES cell lines *in vitro*

**Figure 2:**
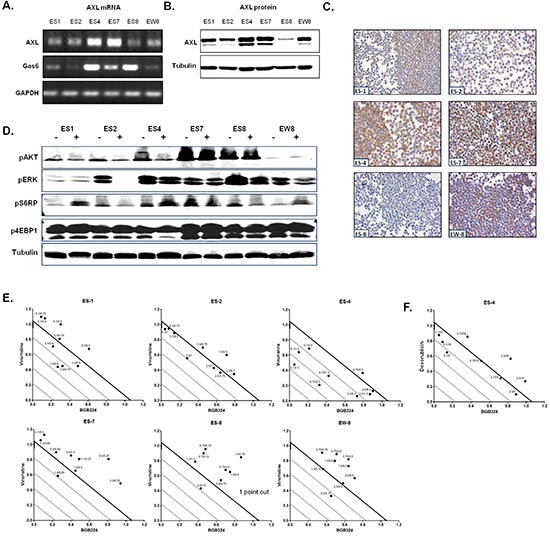
ES cell line characteristics and effects of BGB324 and combined therapies *in vitro* **(A)** AXL and Gas6 mRNA expression in ES cell lines as determined by RT-PCR. GAPDH served as a loading control. **(B** and **C)** AXL protein expression as determined by Western Blot (B; AXL = 138 kDa, Tubulin = 52 kDa) or IHC **(C)**. For Western Blot analysis, tubulin served as a loading control. **(D)** Effects of treatment with IC_50_ concentrations of BGB324 on pAKT, pERK, pS6RP and p4EBP1 expression in ES cell lines by Western Blot. − = untreated; + = treated with BGB324 (IC_50_ differs per cell line; 72 h treatment). Loading was equal in all lines for all experiments, one representative tubulin Western Blot is shown. **(E** and **F)** Synergy experiments for BGB324 combined with either vincristine (E) or doxorubicin (F). The x-axis and y-axis, respectively, show the relative concentration of BGB324 and chemotherapy in synergy compared to the concentrations required in monotherapy to obtain a similar effect on ES cell viability (concentration combination/concentration monotherapy). The bold line represents a combination index of 1. Dots below, on, or above the bold line represent synergy, additivity or antagonism, respectively. Numbers next to the dots indicate the concentrations of BGB324 (left; μmol/mL) and chemotherapy (right, ng/mL) used in the combinations. Points out: the combination of some concentrations of inhibitors resulted in antagonistic effects (CI > 1), which cannot be displayed in the graphs because these data points are outside the axis limits. This is indicated in the corresponding graph (ES-8; 1 data point CI = 1.9).

To further investigate whether AXL represents a potential therapeutic target in ES, we examined the effects of AXL-inhibition (BGB324) and Gas6 stimulation in six ES cell lines *in vitro* by MTT assays. All cell lines demonstrated AXL protein and mRNA expression, although expression levels varied (Figure 2A-C). There was a substantial variation in Gas6 mRNA expression between the cell lines (Figure 2A). Similar to ES specimens, AXL protein expression levels reflected AXL mRNA levels in ES cell lines (Figure 2A-C). BGB324 affected cell viability in all ES cell lines in a dose-dependent manner with IC_50_ values ranging from 0.79–2.13 μmol/L (Supplemental Table 2). No correlations between AXL or Gas6 expression and BGB324 responsiveness were found in this assay. The effects of BGB324 on phosphorylated (p)AKT, pERK, pS6RP and p4EBP1 expression are seen in Figure 2D. Not all cell lines demonstrated similar effects. pAKT and pERK levels either decreased or remained similar, pS6RP levels decreased, increased or remained similar and no apparent effects were observed concerning p4EBP1 expression levels. Gas6 stimulation up to 400 ng/mL did not significantly affect cell viability in any of the ES cell lines in this assay (Supplemental Figure 1).

### BGB324 and chemotherapy

Because in the clinic novel anti-cancer therapeutics are usually combined with conventional cytotoxic treatment, and AXL is implicated in chemotherapy resistance as well as in potentiating the effects of chemotherapy in other tumor types, we tested *in vitro* whether BGB324 synergizes with three commonly used chemotherapeutics in ES, all from different classes of chemotherapy: doxorubicin (anti-tumor antibiotic), vincristine (mitotic inhibitor) and cyclophosphamide (alkylating agent) [10, 25]. The sensitivity of individual ES cell lines to doxorubicin, vincristine and cyclophosphamide (4-HC) monotherapies are summarized in Supplemental Table 2, and ES cell lines were relatively more sensitive to vincristine (IC_50_ 0.58 – 1.72 ng/mL) than to doxorubicin (IC_50_ 5.32 – 59.14 ng/mL) and cyclophosphamide (IC_50_ 225 – 541 ng/mL). The high AXL- and Gas6-expressing ES-4 cell line was the most resistant cell line to vincristine and doxorubicin. The high AXL and low Gas6 expressing EW-8 cell line was relatively the most resistant cell line to cyclophosphamide. The addition of vincristine to BGB324 resulted in synergistic effects (CI < 1) in ES-4 at all but one (nearly additive) tested concentrations, and in the other cell lines at some concentrations (Figure 2E). In ES-1, ES-2, ES-7, ES-8 and EW-8 cell lines, the combination of BGB324 with doxorubicin resulted in antagonistic effects (CI > 1 at all tested doses; not shown). However, in the highly doxorubicin-resistant ES-4 cell line, synergistic or additive effects were observed for most tested concentrations (Figure 2F). The combination of BGB324 with cyclophosphamide resulted in moderate antagonistic effects in all cell lines (data not shown).

### Cell migration assays

**Figure 3:**
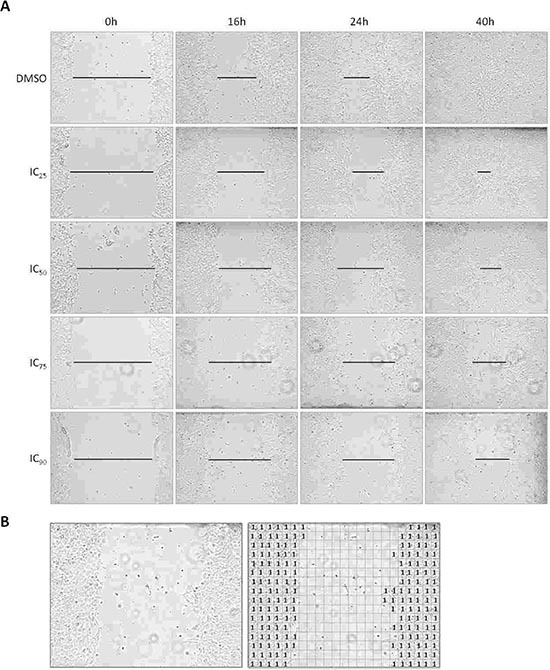
Effects of BGB324 therapy on ES cell migration *in vitro* **(A)** Effects of BGB324 treatment (IC_25_: 1.2 μmol/L, IC_50_: 1.8 μmol/L, IC_75_: 3.0 μmol/L and IC_90_: 4.3 μmol/L) on the migration of ES-8 cells *in vitro* within 40 h. Lines are indicative of wound closure. For quantification of wound closure however, we used a grid to accurately determine the amount of cells that migrated in the gap. All pictures are x10 magnification and representative of three independent experiments. **(B)** Example of quantitative migration analysis in EW-8 cells by placing a grid (23 × 16 square boxes) over the photomicrographs. Left: original photomicrograph. Right: photomicrograph with grid and quantification.

**Figure 4:**
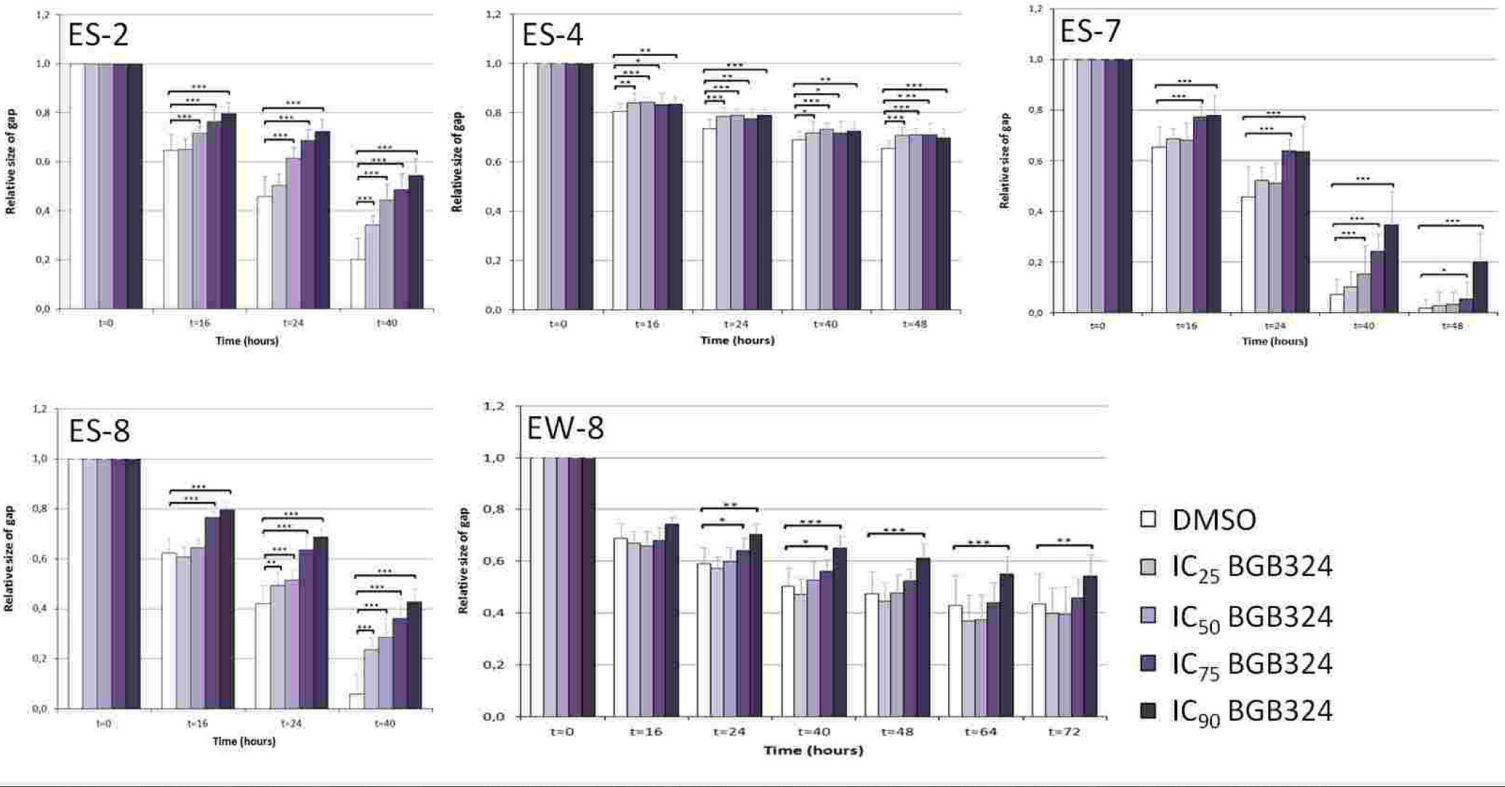
Quantification of the effects of BGB324 on ES cell migration *in vitro* ES cell lines were treated with designated doses of BGB324 (IC_25_, IC_50_, IC_75_ and IC_90_ as determined for each cell line separately) and treated for at least 40 h. Values are presented as mean relative size of gap ± SD. *p < 0.05; **p < 0.01; ***p < 0.001

Because several studies implicated AXL in tumor migration, we investigated whether AXL has similar functions in ES. *In vitro* migration assays were performed with ES-2, ES-4, ES-7, ES-8 and EW-8 cell lines (Figure 3). The ES-1 cell line could not reliably be analyzed in this assay and was therefore excluded from this analysis. Significant inhibition of migration was observed during BGB324 treatment in all tested cell lines (Figure 4). The inhibitory effect on migration was dose-dependent in ES-2, ES-7, ES-8 and EW-8 cell lines, but not in the ES-4 cell line. Significant inhibition of migration was already observed after 16 hours of incubation with IC_75_ and IC_90_ concentrations of BGB324 in all cell lines. The most potent anti-migratory effects were observed in the ES-7 cell line, closely followed by ES-8 and ES-2 cell lines (Figure 4 and Table 3). In ES-8 and ES-2 cell lines, even treatment with the IC_25_ concentration of BGB324 inhibited cell migration at some time points. Although BGB324 significantly inhibited cell migration in ES-4 and EW-8 cells, which was already significant after 16 hours of treatment with the IC_25_ concentration of BGB324 in the ES-4 cell line, the anti-migratory effects in these two cell lines were only minimal. No correlations between AXL or Gas6 expression and BGB324 responsiveness were found in this assay.

**Table 3:** Effects of BGB324 on ES cell migration *in vitro*

Cell line Time point^3^	Effect	Dose BGB324				
		Control	IC_25_	IC_50_	IC_75_	IC_90_
ES-240 h	RGS^1^ ± SD	0.20 ± 0.09	0.34 ± 0.04	0.44 ± 0.06	0.49 ± 0.06	0.54 ± 0.07
	Effect^2^	1.0	1.7	2.2	2.5	2.7
	*p*-value	x	< 0.001	< 0.001	< 0.001	< 0.001
ES-448 h	RGS ± SD	0.65 ± 0.03	0.71 ± 0.04	0.71 ± 0.02	0.71 ± 0.05	0.70 ± 0.04
	Effect	1.0	1.1	1.1	1.1	1.1
	*p*-value	x	< 0.001	< 0.001	< 0.001	< 0.001
ES-748 h	RGS ± SD	0.02 ± 0.03	0.03 ± 0.06	0.03 ± 0.05	0.05 ± 0.07	0.20 ± 0.11
	Effect	1.0	1.5	1.5	2.5	10.0
	*p*-value	x	0.42	0.19	< 0.05	< 0.001
ES-840 h	RGS ± SD	0.06 ± 0.08	0.24 ± 0.05	0.29 ± 0.08	0.36 ± 0.08	0.43 ± 0.05
	Effect	1.0	4.0	4.8	6.0	7.2
	*p*-value	x	< 0.001	< 0.001	< 0.001	< 0.001
EW-872 h	RGS ± SD	0.44 ± 0.12	0.40 ± 0.10	0.40 ± 0.11	0.46 ± 0.08	0.54 ± 0.08
	Effect	1.0	0.9	0.9	1.0	1.2
	*p*-value	x	0.32	0.30	0.54	< 0.01

1 RGS: Relative gap size.

2 Effect: difference in gap size between BGB324-treated cells and control cells, calculated by dividing the mean relative gap size (RGS) of the BGB324-treated cells by the mean RGS of the control cells. A larger effect indicates a larger difference in gap size between BGB324-treated and control cells.

3 Cell lines were treated for at least 40 h, and some cell lines could be evaluated at later time points. Effects are calculated from the latest evaluable time point per cell line.

Migration assays were performed in three separate experiments.

**Figure 5:**
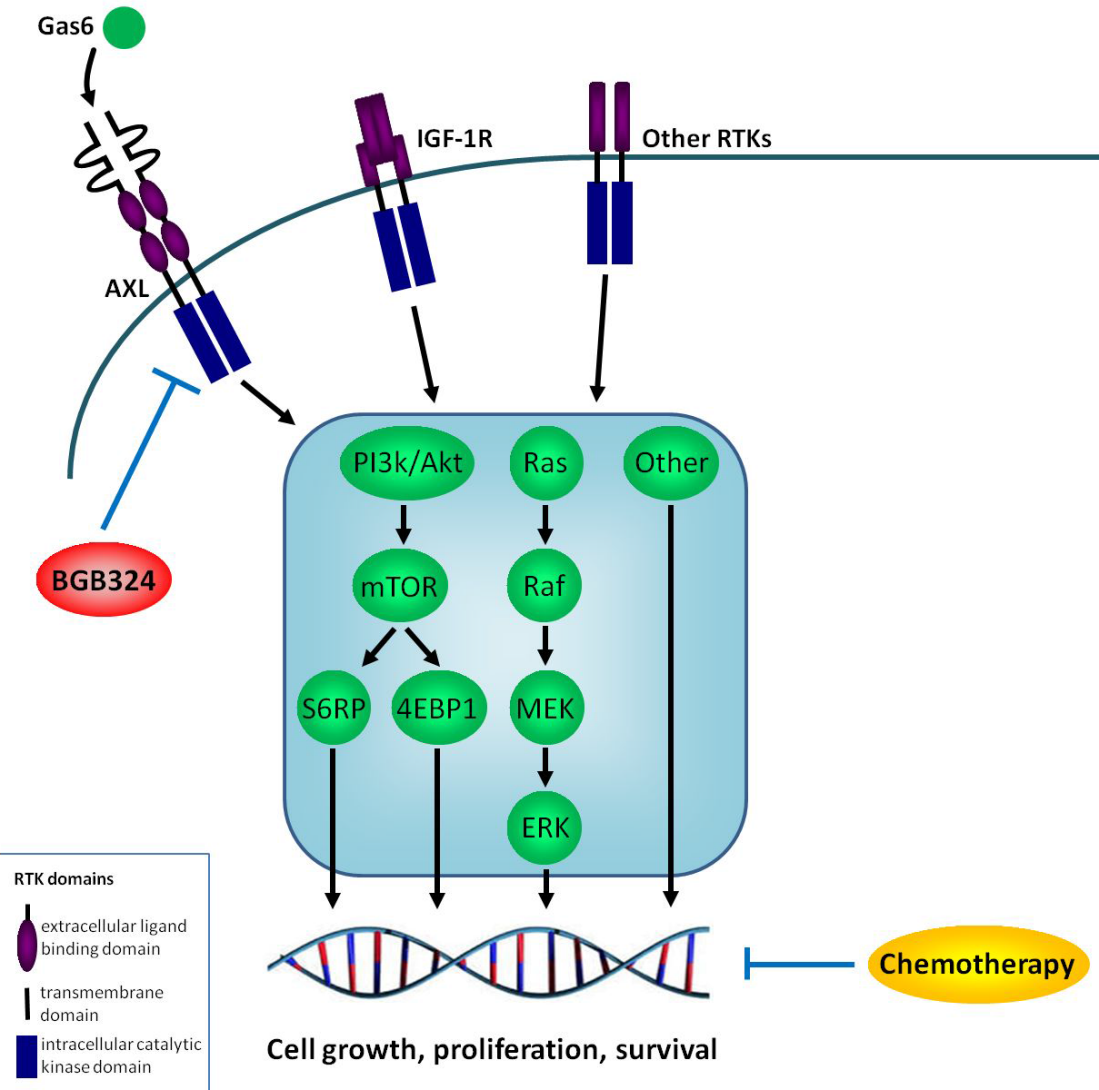
Hypothesized interplay of AXL, Gas6 and BGB324 within the ES cell Hypothesized interplay of AXL, Gas6 and BGB324 within the ES cell, including important downstream signaling pathways and in perspective to chemotherapy and other receptor tyrosine kinases (RTKs). Arrows indicate stimulating effects; blocked lines indicate inhibitory effects. Because this is the first study implicating AXL in ES, we do not know the exact interplay between these factors and cannot give more specific details on potential reciprocal interactions. IGF-1R = Insulin-like Growth Factor-1 Receptor, PI3 k = phosphatidylinositol 3 kinase, mTOR = mammalian target of rapamycin, S6RP = S6 ribosomal protein, 4EBP1 = eukaryotic initiation factor 4E (elF-4E) binding protein-1, Ras = rat sarcoma, Raf = rapidly accelerated fibrosarcoma, MEK = mitogen/extracellular signal-regulated kinase, Erk = extracellular signal regulated kinase.

Figure 5 illustrates the hypothesized interplay of AXL, Gas6 and BGB324 within the ES cell, also in relation to chemotherapy and other RTKs.

## DISCUSSION

AXL is a promising novel therapeutic target in a variety of cancers, such as BC, acute myeloid leukemia (AML), non-small cell lung cancer (NSCLC), pancreatic cancer and prostate cancer [10, 23, 24, 26, 27]. In the present study, we are the first to specifically implicate AXL in ES. We demonstrated that both AXL and Gas6 are abundantly expressed in tumors of ES patients and that high AXL protein expression is an independent prognostic marker of poor OS. We also showed that the specific AXL-inhibitor BGB324 affected ES cell viability and migration in almost all cell lines *in vitro* at clinically achievable doses, highlighting its potential as a novel, druggable therapeutic target in ES.

In this study, AXL was predominantly present in the cytoplasm of ES cells, although membranous, nuclear and vascular expression was occasionally reported as well. This expression pattern closely resembles data in other cancer types [27–30]. Most importantly, high AXL expression levels in primary lesions correlated significantly with a poor OS in ES patients, indicating that AXL-targeting may be of particular interest for patients with the worst prognosis. This is in line with other studies, since a poor OS in ES is the result of an inadequate response to conventional (chemo)therapy and AXL has been specifically implicated in mechanisms of drug resistance [10]. Although the number of patients in our primary tumor cohort was low, and confirmation in a larger, prospective cohort would be mandatory, the correlation between AXL expression and outcome is strong and consistent with a biological hypothesis. Moreover, since various studies reported AXL to be a promising oncological target in BC, and substantially higher AXL expression levels were observed in ES specimens in the present study, this further supports a rationale to target AXL in ES [10, 24, 27, 31]. On top of that, AXL expression was strongly associated with a mesenchymal phenotype across a panel of 643 human cancer cell lines and ES are mesenchymal tumors [32].

In the present study, AXL mRNA expression levels correlated well with protein expression in ES patient samples and cell lines. To further investigate mechanisms underlying aberrant AXL signaling, we screened the AXL RTK domain in several ES patients and in our ES cell lines. However, no aberrations were found, suggesting that ES harbor wild-type AXL, although we cannot exclude aberrations outside the investigated domains. These findings are in line with previous research, since at present no (activating) AXL mutations have been reported in other tumor types as well [29, 33]. The oncogenic potential of AXL is therefore attributed to chronic activation enabled by aberrant receptor expression and autocrine or paracrine ligand stimulation. Interestingly, various studies reported ligand-independent activation of AXL [29, 34]. A similar effect may be present in ES because Gas6 stimulation did not affect ES cell viability, yet BGB324 did affect ES cell viability *in vitro,* suggesting ligand-independent signaling of AXL. We tried to assess phosphorylation of AXL (pAXL) in our ES samples and cell lines, but unfortunately technical difficulties precluded these determinations. Evaluation of downstream pathway activation however demonstrated a decrease in pAKT and pERK levels upon BGB324 treatment in some of the cell lines, which is in line with previous data in other cancer types [24, 26, 32, 35]. The variations in effects on pS6RP expression illustrate the complexity of these signaling cascades and warrant further investigation. Although Gas6 stimulation did not affect ES cell proliferation *in vitro*, BGB324 did affect ES cell viability and impressively reduced cell migration in the majority of ES cell lines. Accordingly, in osteosarcoma and triple-negative BC cell lines, AXL-signaling pathway interference had considerably greater effects on migration than on proliferation *in vitro* [12, 24].

On top of that, there are indications that BGB324 could even be more potent *in vivo* and in ES patients. In addition to tumor AXL expression, vascular AXL was also reported in ES samples. Numerous preclinical and early clinical data show that targeting of vascular mechanisms could be effective in ES and BGB324 was shown to suppress angiogenesis in *in vivo* tumor models [24, 36]. Moreover, the IC_50_ values in our study are nearly identical to those reported in numerous studies in other cancer types (range 0.81 – 3.15 μM) [24, 26, 35, 37, 38]. Some of these studies performed subsequent *in vivo* experiments with the same cell lines as tested *in vitro* and showed effective anti-tumor efficacy of BGB324 at clinically achievable doses [24, 26]. Furthermore, all ES patients showed apparent Gas6 levels to potentially activate AXL. The occasionally reported membranous or nuclear localization of AXL in ES patients may reflect AXL pathway activation, although this requires further investigation.

Although at present no clinical data are available concerning the efficacy and safety of BGB324 in cancer patients, BGB324 was recently evaluated in early clinical safety studies in healthy volunteers with encouraging results. Treatment with BGB324 was established as being safe and well tolerated [23]. Phase Ib clinical trials with this compound will be initiated in the near future for the treatment of aggressive and metastatic cancers, including AML and NSCLC [21, 22]. Other, less specific, AXL-targeting drugs including the multi-kinase inhibitors cabozantinib (VEGFR2, MET and AXL), crizotinib (MET, ALK and AXL), amuvatinib (PDGFR, c-KIT and AXL) and bosutinib (Src, ABL and AXL) have already been tested in cancer patients with promising results and manageable and short-lasting side-effects [39–42]. Crizotinib was even well-tolerated in children [41]. We therefore believe that BGB324 could be a promising drug for future treatment of ES patients. Therapeutic AXL antibodies are also in development with promising *in vitro* and *in vivo* results in several tumor types [43]. For ES however, small molecule inhibitors instead of antibodies may be the drugs of choice since it was recently demonstrated in *in vivo* ES models that a smaller IGF-1R-targeting compound exerted superior tumor penetrating and thus better IGF-1R-targeting properties than a relatively large IGF-1R-targeting antibody [44]. In addition, small molecule inhibitors are capable of inhibiting ligand-independent receptor activation whereas therapeutic receptor-blocking antibodies cannot interfere with this process. Since Gas6 stimulation did not significantly affect ES cell proliferation, yet BGB324 did inhibit ES cell growth, this suggests ligand-independent AXL-signaling which also limits the use of therapeutic antibodies in ES.

Although the results of combined BGB324 and chemotherapy treatments were not straightforward by showing different combined effects with different classes of chemotherapy, the combination of BGB324 with vincristine led to the use of less vincristine in most cell lines to obtain a similar response. This could be an important step towards reducing chemotherapy-induced side-effects. Interestingly, in the high AXL- and Gas6-expressing ES-4 cell line, synergistic or additive effects were observed for most tested concentrations of vincristine and doxorubicin. Because ES-4 was the most resistant cell line to these two types of chemotherapy, this suggests that particularly chemotherapy-resistant, thus poor-prognosis, ES patients might benefit from a combination of BGB324 and chemotherapy. Because in our study, apart from the ES-4 cell line, in all other ES cell lines the combination of BGB324 with doxorubicin resulted in antagonistic effects, this also shows that this particular combination should be handled with caution for ES treatment. The latter also applies to the combination of cyclophosphamide to BGB324, since this led to moderate antagonism in all ES cell lines. Although some studies reported potent effects when combining BGB324 to chemotherapy, including additive effects in combination with doxorubicin, a very recent extensive study investigating the effects of different types of chemotherapy (and other drugs) combined to BGB324 in a large panel of human cancer cell lines stress the importance of selecting the appropriate class of inhibitor for combined treatment regimens [24, 26, 32]. Similar to our present study, different classes of chemotherapy exerted different cytotoxic effects when combined to BGB324 [32]. Antagonism was reported specifically when BGB324 was combined with doxorubicin, while the combination of BGB324 to mitotic inhibitors such as paclitaxel or docetaxel (same class as vincristine) was synergistic. The authors concluded that BGB324 synergistically interacts with anti-mitotic agents and not with doxorubicin to reduce cell viability. Although an alkylating agent such as cyclophosphamide was not included in that study, the platinum drug cisplatin is sometimes grouped with alkylating agents because it kills cells in a similar way [45]. The combination of cisplatin to BGB324 resulted in moderate antagonistic effects in that study, similar to the combination of cyclophosphamide to BGB324 in our present study [32]. Of note, the synergistic interaction between AXL inhibition and anti-mitotic agents was particularly observed in mesenchymal cells in the study of Wilson et al., which further supports its use in ES since these are mesenchymal tumors [32]. From a clinical perspective, AXL-targeting alone or in combination with the appropriate type of chemotherapy could be a promising strategy for ES patients with (early) chemotherapy resistance.

To date, IGF-1R is the most intensively studied RTK target in ES. The lack of activity in high volume malignancies and the observed and not fully elucidated primary and acquired resistance to IGF-1R-targeted therapies in ES patients unfortunately led to the suspension of a lot of planned IGF-1R-targeted research. Because AXL is induced during resistance development to IGF-1R-targeted therapies in rhabdomyosarcomas, which may also be the case in ES, this further supports a rationale to target AXL in ES [18]. Furthermore, Gas6 inhibits IGF-1R signaling [46]. Since Gas6 is ubiquitously present in ES as demonstrated in this study, as well as IGF-1R does, ES may not depend on IGF-1R signaling because this pathway may already be blocked by the abundant levels of Gas6. Gas6 additionally activates AXL, which could mean that AXL-targeting is a more attractive approach to treat ES patients in the future than IGF-1R-targeting. Because evidence is growing towards the conception that the targeting of multiple RTKs is a more effective approach than targeting of a single RTK, and AXL has been implicated in resistance against IGF-1R-targeted therapy, co-targeting of AXL and IGF-1R could also be an effective treatment strategy for ES, which we are currently investigating [4, 18]. This is however still speculative, thus further research on this topic is warranted.

Altogether, we showed for the first time that AXL is a potential novel and druggable therapeutic target in ES. High AXL expression in primary ES appeared to be a strong independent predictor of poor OS. Since the leading cause of ES patient mortality is metastatic disease and BGB324 clearly inhibited ES cell migration *in vitro*, this may offer great potential. Moreover, because AXL is implicated in chemotherapy resistance, and BGB324 chemosensitized chemotherapy-resistant ES-4 cells to vincristine and doxorubicin, the combination of BGB324 to chemotherapy may be of particular interest to ES patients with a poor response to conventional chemotherapy. Our data also stress the importance of selecting the appropriate chemotherapeutic agent for future combination studies, since different classes of chemotherapy exerted different therapeutic effects in combination with BGB324. Although further (*in vivo*) research is warranted, we believe that AXL-targeting is a promising line of inquiry for ES treatment and could have a significant impact on future outcomes for the often young ES patients.

## MATERIAL AND METHODS

### Patient characteristics

Thirty-six tumor samples from 25 patients diagnosed with ES between 1986 and 2009 were retrieved from the files of the dept. of Pathology of the Radboud University Medical Centre (Radboud UMC), Nijmegen, the Netherlands. In all patients the diagnosis was confirmed by ES typical gene fusions and all were treated at the Radboud UMC. The average follow-up was 66 months (range 2–261 months). The study was performed in accordance with the Code of Conduct of the Federation of Medical Scientific Societies in the Netherlands. Patient characteristics are summarized in Table 1.

### Immunohistochemistry (IHC)

In this study we used IHC to specifically investigate AXL protein expression levels and target localization. Selected AXL-positive BC tumor samples and the triple-negative BC cell line MDA-MB-231 served as positive controls and selected AXL-negative BC samples and substitution of the primary antibody by PBS, 1% BSA as negative controls [27, 47]. Control tissues were stained multiple times to verify the reliability and reproducibility of the selected antibody. We also compared AXL expression levels as determined on Western Blot to IHC expression levels of six ES cell lines to further confirm the capacity of IHC to reliably visualize varying AXL expression levels. Subsequently, tissue microarrays (TMAs) were constructed from formalin-fixed paraffin-embedded (FFPE) ES tissue blocks (core size 1 mm) to allow simultaneous examination of ES specimens under identical conditions. Two BC TMAs, consisting of 80 primary BC samples including triple-negative tumors, were stained in parallel. IHC was performed on 4 μm tissue sections. Sections were deparaffinized in xylol and rehydrated through a graded ethanol into water series. Antigen retrieval was performed by microwave heating of slides in a 10 mM sodium citrate buffer, pH 6 for 10 min at 100 °C. Endogenous peroxidase activity was blocked with 3% H_2_O_2_ in distilled water for 10 min at room temperature (RT), and nonspecific binding was prevented by blocking with 20% normal goat serum in phosphate-buffered saline-bovine serum albumin 1% (PBS, 1% BSA) for 30 min at RT. Subsequently, sections were incubated with monoclonal rabbit anti-AXL (1:1000, Cell Signaling Technology, Beverly, MA, USA) antibody in PBS-BSA 1% overnight in a humidified chamber at 4°C. Sections were then incubated with a goat-anti-rabbit biotinylated secondary antibody (1:200 in PBS-BSA 1%, Vector Laboratories, Burlingame, UK) for 30 min at RT followed by an incubation with an avidin-biotinylated horseradish peroxidase complex (ABC) using Vectastain ABC kit (1:100 in PBS-BSA 1%, Vector Laboratories) for 30 min at RT. Next, the catalyzed reporter deposition technique (CARD; 8 min RT) was used to enhance the sensitivity of the staining method followed by a second incubation with ABC for 15 min at RT. Finally, antibody binding was visualized by an incubation for 7 min at RT with 3, 3′-diaminobenzidine (Bright-DAB). Slides were counterstained with haematoxylin, dehydrated and coverslipped.

Slides were scored for AXL expression by two independent observers and consensus cytoplasmic scores were given as follows: 0, no positive cells; 1, low; 2, medium; 3, high staining intensity (> 10% of tumor cells).

### Statistical analysis

Correlations between categorical data were calculated with Fisher's exact test. Wilcoxon signed-rank test was used to compare paired samples. Linear correlation between two continuous parameters was evaluated using Spearman correlation. Kaplan-Meier and log-rank methods were used to draw and evaluate the significance of survival curves. Multivariate Cox regression analysis was used to adjust for other variables after univariate survival analysis. Survival times are presented as mean survival in months ± SD.

### RT-PCR and sequence analysis

Reverse transcription polymerase chain reaction (RT-PCR) of the AXL RTK domain and Gas6 was performed on 29 fresh frozen ES tissue samples (> 50% tumor cells) and 6 ES cell lines. Total RNA was extracted with RNA-Bee (Tel-Test, Friendswood, TX) according to manufacturer's protocol. Then, first strand cDNA synthesis was performed on 1 μg RNA for 1 h at 42°C with random hexamer primers (Promega, Leiden, the Netherlands) and SuperScript II reverse transcriptase (Invitrogen Life Technologies, Breda, the Netherlands). *AXL* RTK domain and *Gas6* genes were subsequently amplified by PCR (Supplemental Table 3). GAPDH was used as a loading control. PCR reactions were performed using AmpliTaq Gold DNA polymerase (Applied Biosystems, Foster City, CA) with 1 μl cDNA and the following programs: 94°C (10 min, all); 92°C (1 min), 62°C (45 sec), 72°C (1 min) for 35 cycles (AXL cDNA sequencing); 94°C (45 sec), 55°C (45 sec), 72°C (1.5 min) for 25 cycles (AXL mRNA expression) or 35 cycles (Gas6 mRNA expression); or 92°C (1 min), 60°C (45 sec), 72°C (1 min) for 25 cycles (GAPDH mRNA expression); and 72°C for 10 min (all). PCR products were analyzed by agarose gel electrophoresis. Subsequently, AXL samples amplified in 35 cycles were submitted to cDNA sequencing using the BigDye Terminator reaction mix, and samples were analyzed on the 3730 DNA Analyzer (Applied Biosystems). Aberrations were confirmed by repeat amplification/sequencing and by consensus among two investigators.

### Western Blot

For Western Blot analysis, protein extracts were purified from ES cell lines. Cells were incubated in ice-cold RIPA buffer containing protease and phosphatase inhibitors and the lysates were centrifuged at 14,000 g at 4°C for 30 min. The protein concentrations of the supernatants were determined with the BCA protein assay system (Pierce Endogen, Rockford, IL, USA). Of each cell line, equal amounts of protein (50 μg) were loaded and run on a 6% Sodium Dodecyl Sulfate-Polyacrylamide Gel Electrophoresis (SDS-PAGE) gel under reducing conditions and subsequently transferred to nitrocellulose membranes. After blocking with blockbuffer (LI-COR Biosciences, Lincoln, NE, USA) in PBS (1:1) at room temperature (RT) for 1 h, membranes were incubated overnight at 4°C with monoclonal rabbit anti-AXL (1:1000), monoclonal rabbit anti-phosphorylated (p) AKT (1:1000), monoclonal rabbit anti-pERK (1:500), polyclonal rabbit anti-pS6RP (1:1000) or monoclonal rabbit anti-p4EBP1 (1:1000). All primary antibodies are from Cell Signaling Technology. Next, the blots were incubated at room temperature for 1 h with a goat-anti-rabbit fluor 680-conjugated secondary antibody (1:5000, AlexaFluor, Invitrogen, OR, USA), incubated for 1 h at RT with monoclonal mouse anti-α-tubulin (1:1000, Abcam, Cambridge, UK) as a loading control and subsequently incubated for 1 h at RT with a goat-anti-mouse fluor 800-conjugated secondary antibody (1:5000, AlexaFluor, Invitrogen). The fluorescent signals were analyzed with the Odyssey Infrared Imaging System (LI-COR Biosciences) and Odyssey Application Software (version 3.0.30).

### Cell lines

ES cell lines (ES-1, ES-2, ES-4, ES-7, ES-8 and EW-8) were generously provided by Peter Houghton of the Pediatric Preclinical Testing Program (PPTP) (Columbus, OH). The presence of EWS/FLI1 gene fusions was confirmed by RT-PCR in all cell lines in our laboratory prior to the initiation of the experiments. Cells were cultured in RPMI-1640 medium (Lonza Benelux BV, Breda, the Netherlands) supplemented with 10% fetal bovine serum (Gibco, Bleiswijk, the Netherlands) and 1% penicillin/streptomycin (Lonza) in a humidified atmosphere of 5% CO_2_/95% air at 37°C.

### Compounds

BGB324 (R428), a well-characterized small molecule inhibitor of AXL, was purchased from Synkinase (Melbourne, Australia). BGB324 is unique in its potent on-target activity and restricted selectivity profile [24]. Doxorubicin and vincristine were purchased (TEVA Pharmachemi B.V., Haarlem, the Netherlands). 4-Hydroperoxycyclophosphamide (4-HC; pre-activated derivative of cyclophosphamide) was purchased as well (D-18864, Niomech, Bielefeld, Germany). Recombinant human Gas6 (rhGas6) was purchased from R&D systems (Minneapolis, USA).

### MTT assays

The effects of BGB324, rhGas6, doxorubicin, vincristine and cyclophosphamide (4-HC) on cell viability were assessed in MTT assays. ES cells were seeded into flat-bottomed 96-well plates at 5,000 cells/100 μl/well and allowed to adhere. Twenty-four hours later, designated drug or ligand doses were added and cells were incubated for 72 h. All drug concentrations and blanks were completed in quadruplicate. Subsequently, 20 μl of 5 mg/ml MTT (Sigma-Aldrich) in PBS was added to each well and cells were incubated for another 3.5 h at 37°C. Afterwards, the medium was carefully removed and the formazan crystals were dissolved in 150 μl of acidified isopropanol solution. Absorbance was read at 560 nm using an ELISA reader. The experiments were repeated in duplo (rhGas6) or triplicate (BGB324, doxorubicin, vincristine and 4-HC) and IC_50_ values were calculated with GraphPad Prism Version 4.00 software.

### Combination indices

To assess drug synergy between BGB324 and doxorubicin, vincristine or cyclophosphamide (4-HC), the combination index (CI) method was used as previously described [48]. In combination experiments, ES cells were treated with three designated doses of BGB324, doxorubicin, vincristine and 4-HC (Supplemental Table 4) and effects on cell viability were assessed by MTT assays. We next identified concentrations of BGB324, doxorubicin, vincristine and 4-HC monotherapies necessary to obtain a similar reduction in cell viability as observed with the combined treatments. Subsequently, CI for the combination treatments was calculated as follows: CI = [Ca,x/ICx,a] + [Cb,x/ICx,b]. Ca,x and Cb,x are the concentrations of drugs A and B used in combination to achieve x% drug effect, ICx,a and ICx,b are the concentrations for single agents to achieve the same effect. A CI < 1 indicates synergy of the combination therapy. A CI equal to or higher than 1 indicates additivity or antagonism, respectively.

### *In vitro* migration assays

Cell wound healing assays were performed as described previously to study cell migration [12]. ES cells were cultured in flat-bottomed six-wells plates at 1.2 – 1.5 × 10^6^ cells/2 ml/well and allowed to adhere. When 80–100% confluence was reached, two slashes were created in the middle of the wells with the tip of a *P*-200 pipette. Cells were rinsed with PBS and replaced with 2 ml media containing designated BGB324 doses (IC_25_, IC_50_, IC_75_ and IC_90_ per cell line as determined with MTT assay (Supplemental Table 5)). After 0, 16, 24 and 40 h incubation, and when possible also after 48, 64 and 72 h incubation, four pictures per well were taken at 10x magnification focused on the gap using the EVOS microscope (AMG/Invitrogen). Wound closure was measured by placing a grid (23 × 16 square boxes) over the pictures and counting the number of positive boxes. A box was considered positive when at least 50% of its surface was covered with (relatively viable) cells (Figure 3B). The degree of wound closure is depicted as relative gap size (RGS = number of negative boxes at any time/number of negative boxes at t = 0 h). Migration assays were performed in three separate experiments. A student's t–test was used to compare differences in wound closure between treatment groups.

## SUPPLEMENTARY TABLES AND FIGUERS


