# Supplementary material for: The role of AXL and the in vitro activity of the receptor tyrosine kinase inhibitor BGB324 in Ewing sarcoma
Source: Oncotarget. 2014 Nov 15;5(24):12753–68. doi: 10.18632/oncotarget.2648 (PMC4350331; doi:10.18632/oncotarget.2648)
Supplement: Supplementary file 1 [file oncotarget-05-12753-s001.pdf]

SUPPLEMENTARY TABLES AND FIGUERS

Supplementary Table S1. Cox multivariate regression analysis of primary tumors scored for AXL

Variable	Hazard Ratio (HR)	*p*-value	95% CI for HR
**AXL high**[1]	141.866	0.021	2.133 – 9434.237
**Gender**	1.849	0.647	0.133 – 25.716
**Age at diagnosis**	0.395	0.651	0.007 – 22.165
**Tumor stage** (at diagnosis)	6.898	0.091	0.732 – 64.987
**Tumor location**	4.113	0.221	0.428 – 39.528

[1]AXL high = AXL score 3
CI = Confidence Interval

Supplementary Table S2. Effect of BGB324, vincristine, doxorubicin and cyclophosphamide (4-HC) monotherapies on ES cell viability *in vitro*

Treatment	Cell line (IC_{50} ± SD)					
	**ES-1**	**ES-2**	**ES-4**	**ES-7**	**ES-8**	**EW-8**
**BGB324** (µmol/L)	0.96 ± 0.08	2.13 ± 0.14	1.97 ± 0.21	0.79 ± 0.03	1.83 ± 0.16	1.84 ± 0.21
**Vincristine** (ng/mL)	0.78 ± 0.09	0.94 ± 0.07	1.72 ± 0.16	0.58 ± 0.03	0.94 ± 0.15	0.71 ± 0.06
**Doxorubicin** (ng/mL)	9.89 ± 1.08	6.84 ± 0.76	59.14 ± 3.75	8.35 ± 1.61	9.42 ± 1.33	5.32 ± 0.39
**4-HC** (ng/mL)	268 ± 39	347 ± 18	277 ± 41	225 ± 59	263 ± 46	541 ± 75

Supplementary Table S3. Primers for RT-PCR analysis of AXL, GAS6 and the GAPDH gene, and for sequence analysis of the AXL RTK domain

Primer	Sequence (5′–3′)	PCR product size (bp)
**AXL-RTKd-1F**	TGTAAAACGACGGCCAGTGTCGGACCACTGAAGCTACC	564
**AXL-RTKd-1R**	CAGGAAACAGCTATGACCCATTCAGCATGCAGTTCCTG	
**AXL-RTKd-2F**	TGTAAAACGACGGCCAGTGAAGTTCATGGCAGACATCG	580
**AXL-RTKd-2R**	CAGGAAACAGCTATGACCAGGATAACCTCCACCCTCATC	
**AXL-F**	GGTGGCTGTGAAGACGATGA	303
**AXL-R**	CTCAGATACTCCATGCCACT	
**GAS6-F**	TGCTGTCATGAAAATCGCGG	286
**GAS6-R**	CATGTAGTCCAGGCTGTAGA	
**GAPDH-F**	CTCTGCTCCTCCTGTTCGAC	98
**GAPDH-R**	TGACTCCGACCTTCACCTTC	

*Primers for sequencing of the AXL RTK domain contain a M13 sequence (underlined) introduced as a site to initiate sequencing.
Abbreviations: F: Forward; R: Reverse; RTKd: Receptor tyrosine kinase domain.

Supplementary Table S4. Doses of BGB324, vincristine, doxorubicin and cyclophosphamide (4-HC) for combination experiments

Treatment	Doses used in combination experiments					
	**ES-1**	**ES-2**	**ES-4**	**ES-7**	**ES-8**	**EW-8**
**BGB324** (µmol/L)	0.1, 0.5 and 1.0	0.1, 1.0 and 2.5	0.1, 0.75 and 2.0	0.1, 0.5 and 1	0.75, 1 and 2	0.75, 1 and 2
**Vincristine** (ng/mL)	0.5, 0.75 and 0.9	0.5, 0.75 and 1.0	0.5, 1.0 and 2.5	0.25, 0.5 and 0.65	0.5, 0.75 and 1.0	0.5, 0.6 and 0.75
**Doxorubicin** (ng/mL)	5.0, 7.5 and 12.5	2.5, 5.0 and 7.5	5.0, 15.0 and 50.0	2.5, 5.0 and 10.0	5.0, 7.5 and 10.0	4.0, 5.0 and 6.0
**4-HC** (ng/mL)	100, 200 and 300	100, 250 and 400	100, 200 and 400	50, 100 and 250	150, 250 and 400	250, 400 and 750

Supplementary Table S5. IC_{25}, IC_{50}, IC_{75} and IC_{90} values per cell line as determined by MTT assay

BGB324 (µM)

	IC25	IC50	IC75	IC90
**ES-1**	0.42	0.96	1.99	3.33
**ES-2**	0.75	2.13	3.44	4.38
**ES-4**	0.39	1.97	3.23	4.44
**ES-7**	0.41	0.79	1.59	2.27
**ES-8**	1.23	1.83	3.02	4.31
**EW-8**	0.96	1.84	3.36	4.43

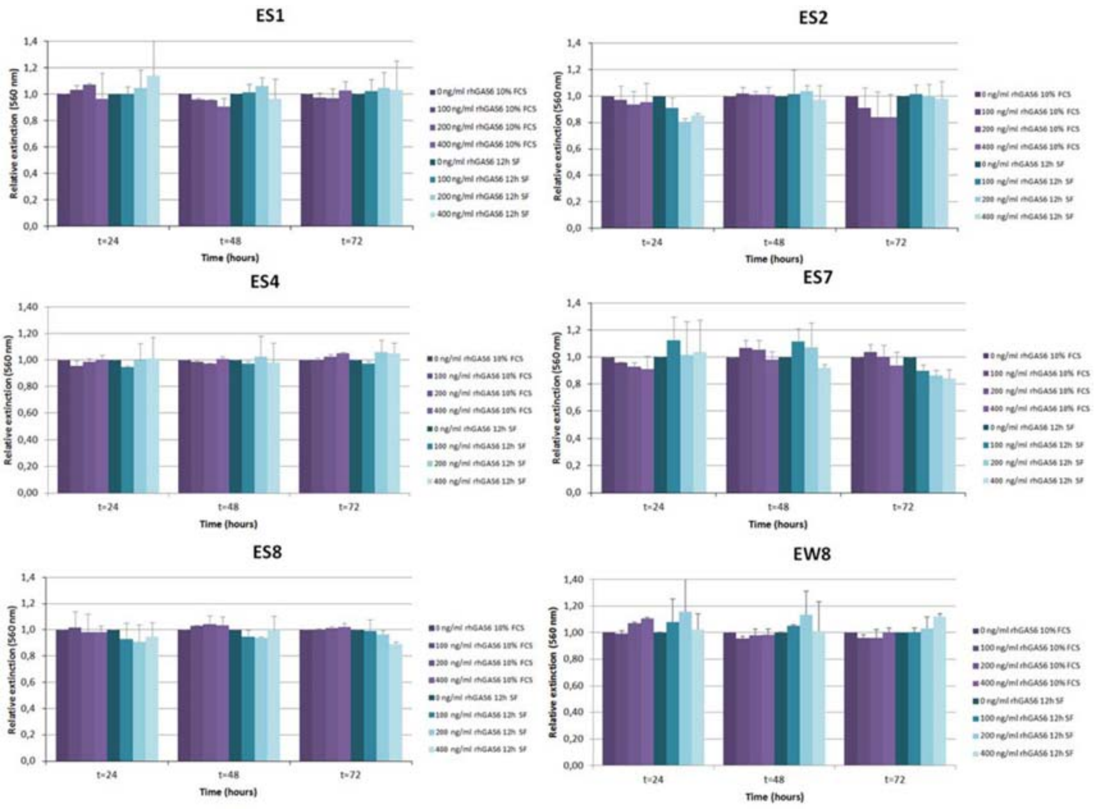

Supplemental Figure S1: Effects of GAS6 stimulation on ES cell proliferation *in vitro*. ES cell viability was determined 24, 48 and 72 h after stimulation with 100, 200 or 400 ng/mL rhGAS6 in the presence or absence (12 h) of 10% FCS. Values are presented as the mean value from three separate experiments in duplo ± SD. SF = serum free.
